# Supplementary material for: Novel Blood Collection Tubes Improve Sample Preservation in a Multicenter Study in Thailand
Source: Diagnostics (Basel). 2025 Sep 20;15(18):2398. doi: 10.3390/diagnostics15182398 (PMC12468855; doi:10.3390/diagnostics15182398)
Supplement: Supplementary file 1 [file diagnostics-15-02398-s001.zip › diagnostics-3855555-supplementary.pdf]

Supplement Table S1

List of biochemical tests and characteristics of automated analyzers used to validate at five clinical laboratories.

Hospital	Direction	Tubes	Parameter	Automated chemistry analyzer		
				**Model**	**Company**	**Country**
Hospital 1 (1,400 beds)	North	Innomed 1	GLU, CHOL, TG, HDL, LDL, BUN, CRE, UA, AST, ALT, ALP, TBIL, DBIL, ALB, TP, LDH, Na^+, K^+, Cl^-, HCO_{-3}	Cobas 758	Roche	Switzerland
		Innomed 2	GLU, CHOL, TG, HDL, LDL, BUN, CRE, UA, AST, ALT, ALP, TBIL, DBIL, ALB, TP, LDH, Na^+, K^+, Cl^-, HCO_{-3}			
Hospital 2 (120 beds)	Southern	Innomed 1	GLU, CHOL, TG, HDL, LDL, BUN, CRE, UA, AST, ALT, ALP FT3,FT4,PSA CEA	Beckman coulter AU5800	Beckman	USA
Hospital 3 (320 beds)	Central	Innomed 1	GLU, CHOL, TG, HDL, LDL, BUN, CRE, UA, AST, ALT, ALP, TBIL, DBIL, ALB, TP, LDH, Na^+, K^+, Cl^-, HCO_{-3}	Mindray Model CL-6000i	Mindray	China
		Innomed 2	TSH, FT3, FT4			
Hospital 4 (1,015 beds)	Central	Innomed 1	GLU, CHOL, TG, HDL, LDL, BUN, CRE, UA, AST, ALT, ALP, TBIL, DBIL, ALB, TP, LDH, Na^+, K^+, Cl^-, HCO_{-3} TSH, FT3, FT4	Beckman coulter AU 480	Beckman	USA
Hospital 5 (650 beds)	Central	Innomed 1	GLU, CHOL, TG, HDL, LDL, BUN, CRE, UA, AST, ALT, ALP, TBIL, DBIL, ALB, TP, LDH, Na^+, K^+, Cl^-, HCO_{-3}	Abbott Accelerator a3600	Abbott	USA
